# EBV and HIV-Related Lymphoma

**DOI:** 10.4084/MJHID.2009.032

**Published:** 2009-12-29

**Authors:** Michele Bibas, Andrea Antinori

**Affiliations:** Clinical Department, National Institute for Infectious Diseases “Lazzaro Spallanzani”, IRCCS, Rome, Italy

## Abstract

HIV-associated lymphoproliferative disorders represent a heterogeneous group of diseases, arising in the presence of HIV-associated immunodeficiency. The overall prevalence of HIV-associated lymphoma is significantly higher compared to that of the general population and it continues to be relevant even after the wide availability of highly active antiretroviral therapy (HAART) (1). Moreover, they still represent one of the most frequent cause of death in HIV-infected patients. Epstein–Barr virus (EBV), a γ-Herpesviruses, is involved in human lymphomagenesis, particularly in HIV immunocompromised patients. It has been largely implicated in the development of B-cell lymphoproliferative disorders as Burkitt lymphoma (BL), Hodgkin disease (HD), systemic non Hodgkin lymphoma (NHL), primary central nervous system lymphoma (PCNSL), nasopharyngeal carcinoma (NC). Virus-associated lymphomas are becoming of significant concern for the mortality of long-lived HIV immunocompromised patients, and therefore, research of advanced strategies for AIDS-related lymphomas is an important field in cancer chemotherapy. Detailed understanding of the EBV lifecycle and related cancers at the molecular level is required for novel strategies of molecular-targeted cancer chemotherapy The linkage of HIV-related lymphoma with EBV infection of the tumor clone has several pathogenetic, prognostic and possibly therapeutic implications which are reviewed herein.

## Epidemiology of AIDS related lymphomas:

Registry linkage studies in the pre-highly active antiretroviral therapy (HAART) era found that the incidence of high grade B-cell non-Hodgkin’s lymphoma (NHL) in HIV-infected individuals was 60–200 times higher than that in HIV-uninfected persons. The introduction of HAART during the mid-1990s has been associated with a fall in incidence of opportunistic infections and AIDS-associated malignancies, including NHL^1^,^2^.

Within the French Hospital Database on HIV Infection (FHDH), the incidence of systemic NHL has decreased between 1993 and 1994 and between 1997 and 1998, from 8.6 per 1,000 to 4.3 per 1,000 person-years, respectively 3; the incidence in the same cohort was 2.8 per 1,000 person-years in 2006. This is consistent with reports of decreased incidence of HIV-related NHL in the post HAART era from the U.K., Australia, California^4^. Nevertheless, the incidence ratio of NHL still remains relatively high in HIV-infected patients (5–6). On the contrary, the incidence of PCNSL has dramatically decreased since the introduction of HAART^4^. Concerning HD, the relative risk is increased, ranging from five- to 25-fold compared to that of the general population^7^,^8^,^9^.

Approximately 1–6% of HIV infected patients develop lymphoma each year. In 2006 the World Health Organization estimated 39.5 million people were living with HIV and that during that year there were 4.3 million new infections with 65% of these occurring in sub-Saharan Africa. Major increases were also seen in Eastern Europe and Central Asia, where it appears that infection rates have risen by more than 50% since 2004. Many of those with retroviral infection will either have limited access to HAART or will be unaware of their HIV status. Therefore the incidence of HIV-associated lymphomas will most likely increase globally in the years to come 10,11.

## Categories of HIV-associated lymphoma:

The WHO (12) classification of lymphoid neoplasms categorises (Table 1) the HIV-associated lymphomas into:

1. Those also occurring in immunocompetent patients, as Burkitt and Burkitt –like lymphomas, Diffuse large B-cell lymphomas Centroblasti and Immnunoblastic (including primary CNS Lymphoma), Extranodal marginal zone lymphoma of Malt Type, Peripheral T-cell lymphoma, Classical Hodgkin lymphoma (80% of all HIV lymphomas);
2. Those occurring more specifically in HIV-positive patients as Primary Effusion Lymphoma^4^ and Plasmablastic Lymphoma of oral cavity type and other variants (3%);
3. Those also occurring in patients with other forms of immunosuppression as Polymorphic B-cell lymphoma (PTLD-like) (5% of all HIV lymphomas).

## Immunodeficiency and pathogenesis of lymphomas in HIV-infected individuals:

HIV is a lentivirus of the retrovirus family, and thus integrates into host chromosomal DNA using a DNA intermediate. It has been generally believed that integration of HIV is a random process, and therefore this process is not in itself oncogenic^13^. Accordingly with this theory is the fact that Southern blot analysis of HIV-associated lymphomas has failed to detect HIV sequences 14, with rare reports of clonal integration restricted to T-cell neoplasms^15^. Although the neoplastic cells are not themselves infected with HIV in most cases, in vitro evidence suggests that HIV does have transforming properties. Laurence and Astrin showed that HIV infection of B-cell lines derived from EBV-seropositive individuals led to B-cell immortalisation, dysregulation of MYC, and activation of EBV^7^. Certain HIV gene products, particularly Tat, have been implicated as potentially oncogenic in their role as transactivators of cellular genes, such as IL6 and IL10 7. Tat protein can more directly interfere with cell cycle control by interaction with the regulatory protein Rb2/p130^8^. This role of the Tat protein has been proposed as a significant factor in the pathogenesis of HIV-related Burkitt lymphoma^8^.

The predominant contribution of HIV to lymphoma pathogenesis is believed to be through indirect mechanisms. The increased risk for lymphoma among HIV-infected individuals appears related to multiple factors, including duration and degree of immunosuppression, induction of cytokines leading to B-cell proliferation, and opportunistic infections with oncogenic herpesviruses such as EBV and HHV8^14^.

HIV-associated malignancies are commonly considered to be the result of diminished immune surveillance against viruses and virus-infected tumor cells. The beneficial effects of HAART on these tumors have therefore been interpreted as the result of drug-mediated HIV suppression and immune reconstitution.

This is supported by several findings. For example, EBV load is increased in patients before development of B-cell lymphoma, whereas specific immune responses against the virus are decreased^16^,^17^,^18^. The relative risk of AIDS-associated malignancies increases progressively as a function of the progressive decline of CD4+ T-cell counts^19^. Nevertheless, the relation between immune deficiency and tumor development is not straightforward.

In fact, only certain types of AIDS-associated tumors arise in immunodeficient patients. In particular, NHL subtypes including Immunoblastic lymphomas and PCNSL, along with Burkitt’s-like lymphomas, typically develop in patients with very low CD4+ T-cell counts. On the other hand, the incidence of other NHL subtypes such as Centroblastic Diffuse Large-cell Lymphomas, along with classic Burkitt’s Lymphoma, Hodgkin’s disease, cervical cancer and, most notably, Kaposi’s sarcoma, increases in patients who have significantly higher CD4+ T-cell numbers^9^,^20^,^21^.

The overall risk of tumour development is very high in HIV-infected individuals, but the relative increase in tumor risk with stepwise decreases in CD4+ T-cell counts is only marginal^22^. It has been observed that the risk of tumor development increases steeply as CD4+ T-cell counts decline below a certain threshold, nevertheless, once below this threshold, cancer risk becomes less dependent on further CD4+ T-cell loss^19^.

However, evidence indicates that this hypothetical CD4+ T-cell count threshold can be very high in certain individuals. In particular, in HIV-infected homosexual men, the incidence rate of Kaposi’s sarcoma increases by more than 1000-fold before a consistent CD4+ T-cell decline^20^. So, CD4+ T-cell loss and consequent immune deficiency cannot fully explain the increased incidence of certain malignancies in HIV-infected individuals. Indeed, several recent studies show that immune activation causes and precedes the development of immune deficiency in HIV infection^23^,^24^,^25^. Sustained and uncontrolled HIV replication leads to continuous antigenic stimulation and to chronic T-cell activation and proliferation, which, in turn, generates a continuous drain of naive and memory T cells that become activated, proliferate, die by apoptosis or re-enter the pool of memory T cells. However, this exhausts the pool of naive T cells, impairing the capacity to mount antigen-specific immune responses^22^,^23^,^24^,^25^.

Several other studies also indicate that immune activation, rather than immune deficiency, is the key factor in the initiation of B-cell lymphomas. In particular, AIDS-associated B-cell lymphomas are described to be preceded by chronic antigen dependent B-cell stimulation leading to a persistent and generalized lymphadenopathy that, in turn, promotes the clonal expansion of pre-neoplastic antigen-specific B-cell populations^26^,^27^.

Furthermore, an increased EBV load precedes the development of B-cell lymphoma^17^, whereas extracellular Tat increases B-cell proliferation and induces B-cell lymphomas in mice^26^,^27^.

## The role of EBV:

Regarding EBV, the percentage of cases within each histotypes with EBV viral infection is variable, ranging from 60% to 100%. In contrast to other lymphomas, a high frequency of EBV association has been shown in HL (80%–100%) tissues from HIV-infected people and the EBV-transforming protein, EBV-encoded latent membrane protein-1 (LMP-1), is expressed in virtually all HIV-HL cases^28^,^29^. On this basis, HL in HIV-infected persons appears to be an EBV-driven lymphoma^30^.

The spectrum of lymphomas occurring in HIV-infected patients includes pathologic subtypes displaying specific association with distinct viruses. BL and DLBCL-IB with plasmacytoid differentiation are often HIV associated and closely linked to EBV infection.

The HIV-associated DLBCL-IB is distinct from other large cell lymphomas occurring in both HIV-seropositive and -seronegative patients because HIV-associated DLBCL-IB lymphomas display a plasma cell–related phenotype.

Most HIV-associated lymphoproliferative disorders, including primary central nervous system lymphoma, systemic DLBCL IB-plasmacytoid, PEL and its solid variant, and PBLs of the oral cavity type, display a phenotype related to plasma cells and are linked to EBV infection.

## Burkitt lymphoma:

Among EBV-positive high-grade B cell Lymphomas, Burkitt Lymphoma (BL) occupies a particular position as being the tumor type in which EBV was discovered. Burkitt and Burkitt-like/atypical Burkitt lymphomas make up the largest group of HIV-associated non-Hodgkin lymphomas, comprising up to 35–50% of these neoplasms in some studies^31^.

Classification of these lymphomas in the HIV setting follows the same diagnostic criteria as are used in the general patient population. That is, a diagnosis of Burkitt or Burkitt-like lymphoma requires a medium-sized CD10-positive B-cell population with a high proliferative rate and demonstration of a translocation involving the MYC gene^12^. Peripheral blood involvement is less common in HIV-infected patients compared to HIV-negative patients with Burkitt lymphoma, although it can occur^12^,^31^,^32^. Burkitt lymphoma occurring in the HIV setting is characterised by multiple genetic lesions, with the relative significance of each in the pathogenesis of this lymphoma unknown. In addition to the translocation involving MYC, point mutations in regulatory regions associated with MYC and within the TP53 tumour suppressor gene are common^12^.

In the context of HIV infection, EBV-encoded RNA (EBER) can be detected by in situ hybridisation in tumor cells in about 30% of Burkitt lymphomas, 50–70% of Burkitt lymphomas with plasmacytoid differentiation, and 30–50% of Burkitt-like lymphomas.

Similarly to sporadic or epidemic forms of Burkitt lymphoma, in HIV-associated EBER-positive disease the viral oncogenes LMP-1 and EBNA-2 are not expressed (Table 2, Table 3).

Although not essential in the pathogenesis of BL, EBV supports tumor development. EBNA-1, a viral protein required for the replication and maintenance of the latent viral episomal DNA, is found consistently in BL cells^33^. The presence of latent EBV in BL cells has been shown to promote genetic instability (34), suggesting a mechanism by which latent EBV could contribute to genetic alterations required for the development of BL.

This is in contrast to EBER-positive immunoblastic DLBCL and PEL, which do show expression of these EBV-associated viral oncogenes. Thus EBV may not play the same role in oncogenesis in these different types of lymphoma. It is interesting to note that although Burkitt lymphoma is common in HIV-infected patients, it is not associated with other forms of immunosuppression.

This may indicate that the oncogenic properties of HIV itself play a greater role in pathogenesis in this highly proliferative tumour compared with EBV or that there are other mechanisms. Dysregulation of cell cycle proteins has been implicated in the development of Burkitt lymphoma. Inactivating mutations of the tumour suppressor gene RBL2 (Rb2/p130) are frequently found in endemic Burkitt lymphoma, and are also found in sporadic cases^35^.

By contrast, in HIV-associated cases, abnormal overexpression of wild-type RBL2 is seen. This finding, in conjunction with studies indicating that the function of Rb2/p130 in the control of the G0/G1 transition can be negated by physical interaction with the Tat protein of HIV-1, may suggest a direct role for HIV proteins acting synergistically with MYC activation in the pathogenesis of Burkitt lymphoma^36^.

## Diffuse large B-cell Lymphoma:

As in the HIV-negative setting, the category of HIV associated DLBCL is a clinically and pathologically heterogeneous group Lymphomas with a predominance of centroblasts have been termed centroblastic DLBCL, whereas those with greater than 90% immunoblasts/plasmablasts have been termed immunoblastic DLBCL.

These two general morphological subtypes show correlation with certain clinical features and molecular profiles. The subtypes occur with approximate equal frequency in HIVinfected patients, with the relative frequency of centroblastic DLBCL increasing and that of immunoblastic DLBCL decreasing in recent years due to advances in HIV therapy. Centroblastic DLBCL occurs in the setting of mild immunosuppression, has a low frequency of EBV positivity (30–40%) without expression of LMP-1, shows a germinal centre B-cell phenotype (expression of CD10 and BCL6, and lack of expression of CD138 and MUM1), and frequently shows rearrangements of the BCL6 gene.

In contrast, immunoblastic DLBCL usually occurs in the context of severe immunosuppression, has a high frequency of EBV positivity (80–90%) with frequent expression of LMP-1 and EBNA-2, shows a non-germinal centre B-cell/activated B-cell phenotype (lack of expression of CD10 and BCL6, expression of CD138 and MUM1), and lacks rearrangements of BCL6^35^ (Table 2, Table 3).

The transforming EBV protein LMP-1 is frequently expressed^37^,^38^. LMP-1 plays a crucial role in the transformation of B-lymphocytes by EBV^40^. Thus, LMP-1 transforms rodent fibroblasts^40^ transgenic mice that express LMP-1 in B cells show increased development of B-cell lymphomas^41^ and LMP-1 deletion mutants of EBV are compromised in their ability to immortalize human primary B cells^42^. LMP-1 activates the NFkB as well as the JNK and p38 pathways (39,40,41), by recruiting cellular TRAF 1–3 and TRADD molecules to 2 short sequence motifs, CTAR-1 and CTAR-2, respectively, in the cytoplasmic domain of the LMP-1 molecule^43^,^44^,^45^.

In B cells, LMP-1 increases the expression of the antiapoptotic proteins A20 and bcl-2, the adherence molecule CD54/ICAM-1, the cell-cycle regulator p27Kip,71 and many others^46^.

In DLBCL, expression of LMP-1 correlates inversely with the expression of BCL6, a marker for germinal center B cells, suggesting that, among DLBCLs, the impact of EBV LMP-1 is likely to be strongest in tumors representing a post–germinal center plasmacytic differentiation profile^47^. In addition, knockdown of LMP-1 in cell lines derived from AIDS-DLBCL results in apoptosis, indicating that this viral oncoprotein plays a role in lymphoma pathogenesis^48^.

EBV-associated DLBCLs have t been considered as EBV-driven lymphoproliferations occurring in the context of a defective T-cell immunity against EBV.^49^ However, unlike EBV-driven lymphoproliferative disease in transplant recipients, which includes monoclonal, oligoclonal, as well as polyclonal B-cell proliferations, DLBCL is always monoclonal. This suggests that, in addition to the effects contributed by EBV LMP-1, additional factors such as genetic damage are likely to contribute to the pathogenesis of AIDS-DLBCL.

## Primary CNS Lymphoma:

Accounting for 15% of HIV-associated lymphomas, PCNSL has a reported incidence of over 1000 times greater than in the non-HIV population^50^. This is most likely a reflection of the brain as a relatively immuno-privileged site. There has been a decline in its incidence since HAART introduction^51^, and it would confirm the strong association of this tumor with severe and prolonged immunosuppression. Clinical presentation results from neurological deficits related to the site of the tumor, with mental state disturbance and seizures more common than in non-HIV PCNSL. Systemic B symptoms are also common^52^,^53^.

These tumors have a tendency to occur late in the course of HIV infection and show EBV association in virtually 100% of the cases^53^. A few studies have reported that detection of EBV in the cerebrospinal fluid of HIV-positive patients with a CNS lesion infers a diagnosis of lymphoma^54^,^55^,^56^. These lymphomas have been reported to express all EBV latent encoded proteins (latency III)^57^, and there are observations consistent with their histogenetic derivation from germinal center-related B cells.^58^ Nevertheless, the exact role of EBV in the pathogenesis of these disorders remains not completely defined (Table 2, Table 3).

Most patients have CD4 counts <50/uL and have multifocal lesions at time of diagnosis. Ocular involvement occurs in up to 20% of cases^59^. Full staging at time of diagnosis is essential to exclude system NHL involving the brain. MRI brain scan has a higher diagnostic yield than CT and is recommended for suspected intracranial masses^60^. Up to 30% of CNS lesions in HIV patients are found to be PCNSL with toxoplasmosis and progressive multifocal leukoencephalopathy making up the remaining cases^60^. The most common histology is immunoblastic variant DLBCL.

Differentiation between PCNSL and toxoplasmosis can be difficult, as both cause ring enhancing lesions with mass effect and oedema (although PCNSL lesions are more likely to be periventricular) and up to 15% false negative rates for toxoplasmosis serology^61^,^62^.

Radionuclide scanning has also been investigated. PCNSL lesions are avid by Thallium^201^ single photon emission CT and fluorodeoxyglucose-positron^18^ emission tomography (FDG-PET), however improve specificity should be combined with PCR and is emerging as an alternative to brain biopsy^63^,^64^. This needs to be further validated and brain biopsy is still the definitive diagnostic procedure, but must be weighed against a mortality rate of 2–3%^64^, particularly in the post-HAART era during which it seems that EBV-DNA detection shows a reduced negative predictive value compared to that of the pre-HAART period.

In the new trials the use of EBV-DNA measurement is used as a surrogate to brain biopsy. Response to therapy may also be monitored with EBV-DNA. There is no standard therapy for PCNSL. Whole-brain radiation (WBRT) achieves CR in up to 50% but this is not translated to increased survival, with median survival no more than 3 months. Deaths are generally related to opportunistic infections due to overwhelming immunosuppression at time of diagnosis. Even though many patients are unable to tolerate the full dose of radiation, the strongest predictors of outcome are performance status and the ability to deliver higher effective radiation doses.

A promising alternative to WBRT was studied in 15 patients using single-agent MTX intravenously at 3g/m2. The mean CD4 count in these patients was 30/uL. Almost 50% had achieved CR with a median survival of 19 months and a relapse rate of only 14%^65^. There is a survival benefit associated with the use of cART after diagnosis^66^, and there is evidence that cART may increase the radio-sensitivity of B cells within the lymphoma^67^,^68^. Given the very limited benefit of current modalities, patients should be referred to clinical trials.

Since there is universal association of EBV in HIV-associated PCNSL, therapeutic options which target the virus have been explored. In this regard it should be noted that EBV-specific allogeneic CTL have been shown to cross the blood brain barrier and induce tumour lysis. In the absence of an available study, either first-line WBRT or alternatively high-dose MTX with the option of WBRT consolidation should be considered. Concomitant HAART therapy to enhance the immune system is critical to successful outcomes

## Classical Hodgkin lymphoma:

HL is the most common type of non-AIDS defining tumor. The risk of developing HL in HIV patients is up to 11–18 times above the general population^69^. It is associated with advanced disease and is more common in the intravenous drug group than in homosexual men. Its hallmark includes aggressive clinical presentation with systemic B symptoms, widespread non-contiguous extranodal lesions and frequent bone marrow involvement (in up to 50% of cases). The morphological patterns are similar to those seen in patients without HIV infection, although with a greater proportion of the subtypes (mixed cellularity, lymphocyte depleted) with less favourable prognosis compared to the general population^70^. As noted above, the greater proportion of mixed cellularity and lymphocyte depleted subtypes appears specifically related to severe immunocompromise in HIV, while HIV-infected patients with modest immunocompromise are more at risk for the development of the nodular sclerosis subtype.

The composition of the reactive inflammatory infiltrate in HIV-associated HL is often characterised by a predominance of CD8-positive T lymphocytes over CD4-positive lymphocytes, by contrast with the background in HL without HIV infection^70^. This finding may simply reflect the depleted peripheral CD4 counts in this patient population. The cytological and phenotypic features of the Hodgkin Reed–Sternberg (HRS) cells in HIV-associated HL are similar to those in non-HIV associated HL. It has been determined that RS cells of all histologic categories of HIV-HD consistently display the BCL-6(-)/syn-1(+) phenotype and thus reflect post-GC B cells^71^.

The HRS cells typically express CD15 and CD30, express CD20 in a minor subset, and lack expression of CD45^70^ In the vast majority of HIV associated HL there is coincident EBV infection. The latent EBV proteins EBNA-1, LMP-1, and LMP2A are expressed in the RS cells, the malignant cell population of this tumor^72^. RS cells are derived from B cells that have passed through the germinal center, as shown by the presence of somatic mutations in the rearranged Ig variable region of their immunoglobulin genes^73^ LMP2A interferes with normal B-cell development, allows BCR-negative B cells to leave the bone marrow/colonize peripheral lymphoid organs^74^, and induces a transcriptome pattern in B cells, which resembles that of HL RS cells^75^. Following EBV infection, LMP2A is essential for the survival and continued proliferation of germinal center B cells lacking a functional B-cell receptor^76^,^77^. LMP2A may therefore promote the survival of “crippled” germinal center B cells and could thus aid their development into RS cells (Table 2, Table 3).

LMP-1 may also induce an “HL-like” transcriptional program in germinal center B cells^78^ Among the cellular genes up-regulated by LMP-1 in HL cells is bmi-1, a polycomb family member known to cause lymphoma in transgenic mice and to down-regulate the ATM tumor suppressor^79^. EBNA-1 was shown to induce CCL-20 secretion in RS cell lines and to thereby promote the migration of regulatory T cells, which could be envisaged to downmodulate EBV-specific T-cell responses^80^.

This association with EBV is considerably stronger than that seen in HL in the non-HIV infected population. HIV-associated HL most often presents at an advanced clinical stage, with B symptoms, frequent extranodal disease, as bone marrow localization, and an aggressive course^81^. Unusual extranodal sites, such as the skin, lung and gastrointestinal tract may be involved^82^. These sites are essentially never involved by HL that is not associated with HIV.

HIV-HL patients have reduced CR rates and survival compared with the HIV negative population. In the early years post-HAART therapy the incidence of HIV-HL appeared to be in decline however two studies showed that the incidence may actually have increased^83^,^84^.

The post-HAART era was also associated with an improvement in survival which was attributed to virological response to antiretroviral therapy and a reduction in HIV-associated mortality^85^. In another study of 47 patients in the post-HAART era, the median survival was not reached compared with 19 months in the pre-HAART era^86^,^87^.

Optimal therapy for HIV-HL has not been defined. Treatment regimes used are similar to those used in HL in the seronegative population^88^,^89^.

## Primary effusion lymphoma (PEL):

PEL is a distinct clinicopathological entity occurring almost exclusively in HIV-infected patients. This lymphoma subtype comprises less than 5% of all HIV-associated NHL. Cases of this type were first described by Knowles et al in 1989^90^, but its distinctive features were not fully recognised until after the identification of the Kaposi sarcoma-associated herpesvirus/human herpesvirus 8 (KSHV/HHV8) in 1994^91^,^92^, PEL is a distinct type of B-cell non-Hodgkin lymphoma (NHL) that presents most frequently in body cavities as lymphomatous effusions without an associated tumor mass.

The tumor cells have large round to irregular nuclei with prominent nucleoli, and abundant deeply basophilic and occasionally vacuolated cytoplasm. These are described as immunoblastic/plasmablastic or anaplastic morphological features. Recent studies have broadened the scope of PEL to include those presenting as a solid tumour mass with or without an associated effusion^93^,^94^,^95^. The so-called “extracavitary” or “solid variant” of PEL most commonly involves the gastrointestinal tract or soft tissue, but can also involve lymph nodes. Some studies have suggested that the extracavitary variant of PEL has a slightly better prognosis when compared with cases presenting with effusion^95^,^96^.

A defining property of PEL is its consistent association with KSHV infection. Most cases are also co-infected by EBV. It is believed that KSHV, rather than EBV, is a driving force in these tumors, as in PEL, at least 5 KSHV viral genes are expressed, which provide proliferative and antiapoptotic signals. In contrast, EBV has a restricted latency pattern of gene expression in PEL, where only EBNA1 and EBERs are expressed^97^. However, the viral oncoprotein LMP-1 is generally not expressed^94^,^95^,^96^,^97^ (Table 2, Table 3).

The immunophenotypic features of PEL often make it difficult to confirm B-cell lineage, as the neoplasm usually lacks expression of most B-cell associated antigens including CD19, CD20, CD79a and immunoglobulins. The most frequently expressed antigens include those associated with activation or plasmacytic differentiation, such as CD30, CD45, EMA, CD71, MUM1, and CD138. Aberrant expression of Tcell associated antigens CD3 and CD7 has been reported^98^,^99^,^100^,^101^,^102^.

B-cell origin of PELs, can be demonstrated by the presence of clonal immunoglobulin gene rearrangements. Evidence points toward a post–germinal center B-cell derivation, as most PELs contain somatic hypermutation of Ig genes as well as frequent somatic hypermutation of the noncoding region of the *BCL6* gene^103^,^104^. Consistent with this notion is the expression of plasma cell markers such as CD138/Syndecan-1. Recently, gene expression analysis of PEL showed features most similar to AIDS immunoblastic lymphoma and multiple myeloma, again indicating a pre–plasma cell or “plasmablastic” profile^105^.

Again, the exact role of EBV has been debated; but the fact that both viruses are detected together in most of the cases suggests that EBV may act as a cofactor in the initiating events (because it can immortalize and transform B cells in vitro and HHV-8 cannot), whereas HHV-8 may be the driving force for the tumor^106^. With or without therapy, PEL is invariably associated with an adverse prognosis. There is limited data on the treatment of PEL. The destruction of local tissue despite aggressive therapy leads to shortened survival^107^.

Chemotherapy and radiotherapy may result in responses but these are seldom durable and survival is generally less than 12 months although a small series suggests the addition of high-dose MTX may improve outcome^107^. Interestingly, a patient treated with a combination of zidovudine and α-interferon (α-IFN) entered into durable remission after only 5 days^108^. Study of the primary tumour cells derived from this patient demonstrated that azidothymidine (AZT) blocked nuclear translocation of NFkB and potentiated the pro-apoptotic effect of a-IFN (which induces another death receptor ligand, TRAIL). Further clinical studies of this combinationare under way. In a murine system sirolimus showed promising activity which was in part mediated by inhibition of IL-10 signaling^108^. Given the relative rarity of this lymphoma, patients should be enrolled in clinical trials where possible. Concomitant administration of HAART is advised and there are several reports of remission of PEL with use of HAART alone.

## Plasmablastic lymphoma of the oral cavity type:

Plasmablastic lymphoma is a distinct type of diffuse large B-cell lymphoma that occurs most often in the oral cavity or jaw of HIV-infected individuals^110^. This rare lymphoma subtype accounts for 2.6% of HIV-related NHL^111^ The first description designated this tumour as a lymphoma of the oral cavity68; however, subsequent reports have described less frequent involvement of extraoral sites such as the anal cavity, gastrointestinal tract, lung, paranasal sinus, skin, spermatic cord, testicle, bone and lymph nodes^112^–^118^.

Regardless of the site of occurrence, plasmablastic lymphoma shows similar morphological and phenotypic features. The neoplastic cells are intermediate to large in size, with round nuclear contours and occasional multinucleation. Plasmacytic differentiation is usually apparent, with a cytological spectrum including a minor population of small plasmacytoid cells with condensed chromatin ranging to large cells with dispersed chromatin, prominent central nucleoli and abundant basophilic cytoplasm with a paranuclear hof^112^,^113^,^114^. The neoplastic population generally expresses CD45 and plasmacytic markers such as CD138, EMA and MUM1, and usually lacks expression of pan-B-cell antigens such as CD20 and PAX5.1^110^,^116^. In early reports, slightly more than 50% of cases were EBER positive as shown by in situ hybridisation studies^110^ in more recent series all cases of plasmablastic lymphoma have been shown to be EBER positive^115^, 73 EBER-positive cases generally lack expression of EBNA2 and LMP-1^115^,^116^ (Table 2, Table 3). HHV8 infection is not implicated in the pathogenesis of plasmablastic lymphoma, with all cases negative for LNA1 when tested by immunohistochemistry. While there is morphological and phenotypic overlap with anaplastic myeloma, extramedullary presentation and frequent EBV infection are distinctive features. A potential role for EBV in the pathogenesis of the disease remains unknown, especially with the highly restricted latency expression pattern. Despite the use of aggressive chemoteraphy and HAART the prognosis remains poor^119^.

## Polymorphic B-cell Lymphoma (PTLD-like):

HIV infection results in a reduction of T-cell immunity similar to that iatrogenically induced in transplant patients. Is not surprising that polymorphic lymphoid proliferations resembling post-transplant lymphoproliferative disorders (PTLD) have been reported in HIV-infected adults and children. According to the WHO classification, they are divided into early lesions (reactive plasmacytic hyperplasia and mononucleosis-like syndrome), polymorphic lesions, monomorphic lesions, and Hodgkin-like lesions^120^. Similarly to PTLD, these infiltrates are often associated with EBV infection. By contrast with HIV-associated lymphoma, these polymorphic infiltrates often show more limited disease distribution, lack oncogene and tumour suppressor gene alterations, and may be polyclonal or show a minor B-cell clone in a polyclonal background. Regression of polymorphic B-cell lymphoma in an HIV-infected patient after anti-retroviral therapy has been reported^121^.

EBV has been linked to most PTLDs, with a near 100% association in the early-occurring cases (within a year) and in PTLD-associated Hodgkin lymphoma^122^. The EBV-negative PTLDs constitute approximately 20% of all cases, have a tendency to late occurrence and have an unknown etiology. Type III latency is exhibited by the EBV-positive B cells in PTLD, although some studies have reported a more restricted latency pattern^123^. The wide expression of the latent EBV-encoded proteins strongly suggests an important role that EBV may play in the oncogenic process (Table 2, Table 3).

The mechanism by which EBV is thought to contribute to the pathogenesis of PTLD is similar to its presumed role in Hodgkin lymphoma. Because approximately 50% of PTLD cases are derived from GC B cells lacking a functional BCR because of certain crippling mutations, and because these cells manage to escape apoptosis despite lacking antigen affinity, it is believed that EBV aids in rescuing these cells from an imminent programmed cell death^124^,^125^. As in Hodgkin cases, LMP1 and LMP2A may replace survival signals induced by activated BCR and CD40 receptors and also activate the NF-κB signaling pathway, inducing proliferation of neoplastic cells. The decreased cytotoxic T-cell surveillance because of immunosuppression in PTLD patients is also believed to greatly facilitate the actions of EBV. The similar role that EBV is thought to play in inducing the survival and neoplastic transformation of infected GC cells in both PTLD and Hodgkin lymphoma, in addition to the near 100% EBV positivity in PTLD-associated Hodgkin lymphoma, has led some investigators to speculate a connection between the 2 diseases and the possibility that EBV infection and its GC effects may be the initiating role in the pathogenesis of both entities^124^.

## Conclusions:

HIV-associated lymphomas represent a particular setting characterizing specific pathogenetic models prevalently driven by EBV and by immunodeficiency. The impact of combined antiretroviral therapy has substantially changed the risk and prognosis of lymphoma in HIV-infected population, as well as the relationship with the natural history of HIV disease. As a consequence of cART, many authors now strongly recommend that patients with lymphoma and HIV infection should be treated as patients with lymphoma of the general population.

In fact, due to the improvement of morbidity and mortality related with cart exposure, more aggressive treatment protocols can be taken into consideration, on the bases of the results in terms of efficacy and tolerability reported in the general population, such as the use of high-dose chemotherapy in combination with PBSC transplantation in HIV-NHL which showed response rates similar to those obtained in HIV-negative patients.

The concurrent use of antiblastic chemotherapy and cART should be considered a potential advantage for tumor prognosis and for reducing risk of toxicities associated to antineoplastic drugs, even though concerns due to drug-drug interactions could be suggested. In perspectives, the molecular and epidemiological linkage between AIDS-related malignancies and EBV-infection suggests that viral gene products would be potential targets for molecular-targeted chemotherapy. Detailed understanding of the EBV lifecycle and related cancers at the molecular level may lead to the development of novel strategies of molecular-targeted cancer chemotherapy to specific viral oncogenes to which the lymphoma cells are addicted, and that will provide therapeutic benefits.

## Figures and Tables

**Table 1.** Classification of HIV-associated lymphomas

**1. Lymphoma also occurring in immunocompetent patients:**
a. Burkitt and Burkitt-like Lymphoma
b. Diffuse large B-cell lymphoma
**i.** Centroblastic
**ii.** Immunoblastic (including primary CNS lymphoma)
c. Extranodal marginal zone lymphoma of Malt type
d. Peripheral T-cell lymphoma
e. Classical Hodgkin Lymphoma
**2. Lymphoma occurring more specifically in Hiv positive patients**
a. Primary effusion Lymphoma
b. Plasmablastic lymphoma of the oral cavity type
**3. Lymphoma also occurring in other immunodeficiency states**
a. Polymorphic B-cell lymphoma (PTLD-like)

**Table 2.** Immunological and EBV status in AIDS-related Lymphomas

**Lymphoma Histology**	**immunodeficiency**	**% in HIV**	**EBV+ Rate**	**Viral cofactor**
Systemic AIDS-Related Lymphomas				
Burkitt Lymphoma	Mild	55		EBV EBER
Classic BL		30	30%	
BL with Plasmocitoid diffent		20	50–70%	
Atipical BL		Less freq	30–50%	
Diffuse Large B-cell lymphoma		30		
Centroblastic type	Mild	20	30–40%	
Immunoblastic type	Marked	10	90–100%	EBV LMP1
AIDS Primary CNS Lymphoma	Marked	< 5	100%	EBV LMP1
Primary effusion Lymphoma	Marked	< 5	90%	EBV
Plasmablastic lymphoma oral cavity		< 5	50%	LMP1
Hodgkin Disease Classical	Marked		100%	LMP1 LMP2A

**Table 3.** Features of EBV-associated Aids-associated B-cell Lymphoma

**Lymphoma**	**EBV %**	**EBV Latency**	**Phenotype**	**Cellular origin of lymphoma cells**
Hodgkin L Classical	100%	Type II	Loss B-cell Phenotype	Pre-apoptotic GC B cells
PCNSL	100%	Type III	BCL6− CD138+ Mum 1+	GC or post GC B cells
Burkitt Lymphoma	55%	Type I	BCL6+ CD10+ CD77+	GC B cell
PEL	90–100%	Type I	Loss B-cell Phen. CD38+	GC or post-GC B cells
DLCL-CB	30%	Type I	BCL6+ CD138− MUM1−	Mostly GC or post-GC B cells
DLCL-IB	90%	Type III	BCL6− CD138+MUM1+	Mostly GC or post-GC B cells
